# Intralesional Rose Bengal in melanoma elicits tumor immunity via HMGB1

**DOI:** 10.1186/2051-1426-3-S2-P408

**Published:** 2015-11-04

**Authors:** Hao Liu, Krithika Kodumudi, Amy Weber, John Robinson, Satoshi Nemoto, Georgina Crago, Timothy McCardle, Erica Royster, Amod A Sarnaik, Shari Pilon-Thomas

**Affiliations:** 1 H. Lee Moffitt Cancer Center & Research Institute, Tampa, FL, USA

## 

Intralesional (IL) therapy is under investigation to treat dermal and subcutaneous metastatic cancer. Rose Bengal (RB) is a staining agent that was originally used by ophthalmologists and in liver function studies. Previously, IL injection of RB induced regression of injected and uninjected tumors in murine models. However, the relevant mechanism is yet unknown. In this study, we used an OVA-expressing B16 melanoma murine model and found that IL RB treatment led to increased tumor-specific T cells with memory characteristics. CD8+ T cell are crucial for tumor-specific response elicited by IL RB. IL RB therapy also increased antigen-specific T cell proliferation and enhanced tumor regression. In addition, IL RB facilitated dendritic cells (DCs) infiltrating lymph nodes draining from tumor. Incubation of melanoma cells with RB led to necrosis and the release of High Mobility Group Box 1 (HMGB1), which activated DCs via up-regulation of CD40 expression. The blockade of HMGB1 significantly reduced the antigen-presenting ability of DCs. To determine whether this mechanism was relevant in patients treated with IL RB, we performed a pilot clinical study in melanoma patients (NCT01760499). IL RB led to tumor regression in both RB-injected and uninjected lesions, associated with an increase in circulating T cells. Increased tumor-specific response was found from those circulating T cells of 5 out of 7 tested patients after IL RB treatment. HMGB1 levels in patient sera were also elevated. Together, these results reveal a clinically relevant immunoadjuvant pathway triggered by tumor cell death secondary to ablation with RB.

## Trials Registration

ClinicalTrials.gov identifier NCT01760499.

